# Alpha transcranial alternating current stimulation reduces depressive symptoms in people with schizophrenia and auditory hallucinations: a double-blind, randomized pilot clinical trial

**DOI:** 10.1038/s41537-022-00321-0

**Published:** 2022-12-24

**Authors:** Mengsen Zhang, Rachel B. Force, Christopher Walker, Sangtae Ahn, L. Fredrik Jarskog, Flavio Frohlich

**Affiliations:** 1 Department of Psychiatry, University of North Carolina, Chapel Hill, NC USA; 2 Carolina Center for Neurostimulation, University of North Carolina, Chapel Hill, NC USA; 3 School of Electronic and Electrical Engineering, Kyungpook National University, Daegu, South Korea; 4 Neuroscience Center, University of North Carolina, Chapel Hill, NC USA; 5 Department of Cell Biology and Physiology, University of North Carolina, Chapel Hill, NC USA; 6 Department of Biomedical Engineering, University of North Carolina, Chapel Hill, NC USA; 7 Department of Neurology, University of North Carolina, Chapel Hill, NC USA

**Keywords:** Neural circuits, Schizophrenia, Biomarkers

## Abstract

People with schizophrenia exhibit reduced alpha oscillations and frontotemporal coordination of brain activity. Alpha oscillations are associated with top-down inhibition. Reduced alpha oscillations may fail to censor spurious endogenous activity, leading to auditory hallucinations. Transcranial alternating current stimulation (tACS) at the alpha frequency was shown to enhance alpha oscillations in people with schizophrenia and may thus be a network-based treatment for auditory hallucinations. We conducted a double-blind, randomized, placebo-controlled pilot clinical trial to examine the efficacy of 10-Hz tACS in treating auditory hallucinations in people with schizophrenia. 10-Hz tACS was administered in phase at the dorsolateral prefrontal cortex and the temporoparietal junction with a return current at Cz. Patients were randomized to receive tACS or sham for five consecutive days during the treatment week (40 min/day), followed by a maintenance period, during which participants received weekly tACS (40 min/visit) or sham. tACS treatment reduced general psychopathology (*p* < 0.05, Cohen’s d = −0.690), especially depression (*p* < 0.005, Cohen’s d = −0.806), but not auditory hallucinations. tACS treatment increased alpha power in the target region (*p* < 0.05), increased the frequency of peak global functional connectivity towards 10 Hz (*p* < 0.05), and reduced left-right frontal functional connectivity (*p* < 0.005). Importantly, changes in brain functional connectivity significantly correlated with symptom improvement (*p* < 0.05). Daily 10 Hz-tACS increased alpha power and altered alpha-band functional connectivity. Successful target engagement reduced depression and other general psychopathology symptoms, but not auditory hallucinations. Considering existing research of 10Hz tACS as a treatment for major depressive disorder, our study demonstrates its transdiagnostic potential for treating depression.

## Introduction

Alterations in brain oscillations and the temporal coordination of neural activity between brain regions have been associated with a wide range of psychiatric disorders^1^. Rhythmic brain stimulation, such as transcranial alternating current stimulation (tACS), transcranial magnetic stimulation (TMS), and deep brain stimulation (DBS), has been employed to restore physiological brain oscillations and thereby treat symptoms in patients^2–4^.

Schizophrenia is characterized by positive symptoms (e.g., hallucination, delusions, disorganized thinking), negative symptoms (e.g., blunted affect, avolition, social withdrawal), and general psychopathology^5^. The underlying neural mechanisms involve both distributed functional connectivity deficits and more circumscribed hyper/hypoactivity^6^, implicating abnormal oscillatory dynamics at both low frequencies (alpha, theta) and high frequencies (gamma)^7,8^. In particular, auditory hallucinations represent a prevailing positive symptom of schizophrenia^9,10^. They are often associated with hyperactivity in the auditory cortex near the left temporoparietal junction (TPJ) and are thought to reflect abnormal bottom-up activity in the auditory pathway^11–15^. In addition, auditory hallucinations and schizophrenia, in general, are also associated with hypoactivity in frontal regions such as the dorsolateral prefrontal cortex (dlPFC)^11,16–18^, thought to reflect deficits in top-down inhibitory control^19^. Moreover, miscommunication between bottom-up activity and top-down control is thought to underlie the pathology, e.g., top-down activities fail to censor erroneous inputs or internally generated speech (corollary discharges) from the auditory cortex^20–22^. This idea is supported by the observation of reduced frontotemporal functional connectivity^23,24^ and structural connectivity^25^ in people with schizophrenia.

Alpha oscillations are associated with the top-down inhibitory gating of sensory information^26,27^. Schizophrenia is associated with reduced alpha oscillations and altered functional connectivity within the alpha frequency range^17,28–32^. Specific changes to the alpha oscillation in schizophrenia include a lower individual alpha frequency^33^ and reduced alpha power^17,28,34,35^, which can be considered a heritable neuromarker or endophenotype^36–38^. The topography of alpha oscillations was found to shift from posterior to more anterior regions^39^. Further, decreased functional connectivity in the alpha band between the prefrontal cortex and superior temporal cortex has also been reported^29^. These observations suggest that spatially and temporally altered alpha oscillations may contribute to dysregulated top-down control in the frontotemporal network of patients with auditory hallucinations. Thus, the alpha oscillation represents an attractive target for non-invasive brain stimulation in schizophrenia.

tACS is a non-invasive method of brain stimulation and a key tool for investigating the causal roles of neural oscillations in a frequency-specific manner^2,40^. Animal studies have shown that weak oscillating electric fields of comparable strength (<1 V/m), applied either intracranially or transcranially, are able to entrain neuronal activity^41,42^. Simultaneous tACS-electroencephalography (EEG) demonstrated entrainment of endogenous occipital alpha oscillations by 10-Hz tACS^43^. Importantly, entrainment by alpha-tACS can lead to a lasting after-effect of enhanced endogenous alpha power (>1 h for 20 min stimulation)^44,45^. More recently, alpha-tACS was studied as a treatment for psychiatric disorders^3^ such as major depressive disorder^46–48^ and schizophrenia^49,50^. In a recent double-blind clinical trial from our group^49,51^, 10-Hz tACS enhanced alpha power in schizophrenia patients with auditory hallucinations at the end of the course of a 5-day treatment. Although the clinical effect on auditory hallucinations was not statistically significant, alpha power increase in left temporoparietal regions was significantly related to symptom reduction in the group of patients that received verum tACS.

The present study extended the 5-day treatment regimen by adding a two-month maintenance period where participants received weekly sessions of 10-Hz tACS (or sham) together with repeated measurement of symptoms and alpha power. We hypothesized that (1) 5-day consecutive 10Hz-tACS reduces auditory hallucination symptoms, while weekly tACS maintenance extends the duration of symptom improvement, and (2) changes in alpha oscillations and functional connectivity are correlated with the improvement of clinical symptoms. We found that 5-day consecutive 10Hz-tACS, but not weekly maintenance tACS, significantly increased alpha power and altered functional connectivity within the alpha frequency band. We found no significant reduction of auditory hallucination symptoms by tACS. Instead, people with schizophrenia who received 5-day tACS exhibited a decrease in depression and other general psychopathology symptoms that significantly correlated with changes in brain functional connectivity.

## Methods

### Participants

This study was conducted from November 14th, 2017, to January 5th, 2021, at the University of North Carolina at Chapel Hill (ClinicalTrials.gov NCT03221270: “Targeting Auditory Hallucinations With Alternating Current Stimulation,” STILL3) and was approved by the UNC-Chapel Hill Institutional Review Board. Participants were recruited from local clinics affiliated or unaffiliated with the university. The target sample size was 40, which was determined based on our previous pilot clinical trial^49,51^.

### Inclusion and exclusion criteria

All participants were diagnosed with schizophrenia, any subtype, or schizoaffective disorder (confirmed by the Structured Clinical Interview for DSM Disorders-IV^52^) with symptoms present for greater than 1 year, who also met the following criteria: 18–70 years old; experiencing at least three auditory hallucinations per week; clinically stable (no hospitalization in the past 12 weeks); on stable doses of antipsychotic medications (no changes in doses or medication for 4 weeks prior to enrollment). In addition, participants were required to meet the criteria for treatment-resistant auditory hallucinations, defined as having ongoing auditory hallucinations during trials of at least 2 different antipsychotic agents of adequate dose and duration^51,53^. To further define the experience of auditory hallucinations, the variability was limited as eligible participants were interviewed twice over the course of 2 weeks during the screening period and only enrolled if they demonstrated ≤20% change in their Auditory Hallucination Rating Scale (AHRS) scores.

Individuals were excluded from the study for the following criteria: concurrent (within 4 weeks) anticonvulsant medications or daily treatment of benzodiazepines (limited as-needed use that was discontinued more than 48 h prior to a study session was allowed); A DSM-IV diagnosis of alcohol or substance dependence within the past 6 months; positive urine test of cannabis, cocaine, amphetamine, barbiturates, opiates; history of significant head injury or traumatic brain injury, prior brain surgery or any brain devices/implants, history of seizures, unstable medical illness, or pregnancy; non-English speakers.

This study used a Data Safety Monitoring Board (DSMB) through the North Carolina Translational and Clinical Sciences Institute. Bi-annual reviews of blinded data and adverse events were submitted to the DSMB. Study data were collected and managed using REDCap electronic data capture tools^54,55^.

### Study design and blinding procedures

**Fig. 1:**
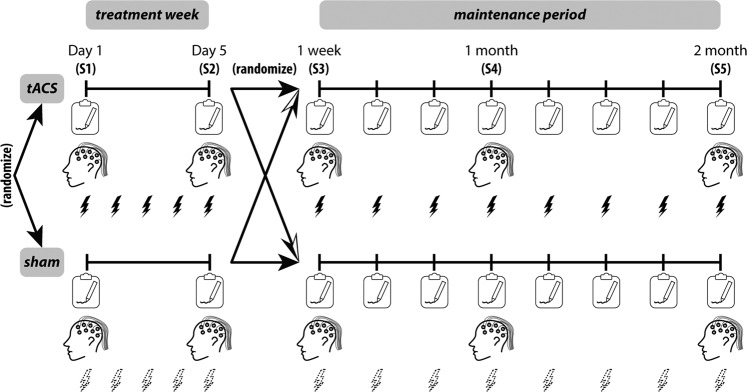
Cross-over design to study the effect of daily tACS treatment and weekly maintenance. The study consisted of two stages: the treatment week (left) and the maintenance period (right). Initially, participants were randomly assigned to the tACS group (upper left) or the sham group (bottom left), who received five consecutive days of daily 10 Hz tACS or sham stimulation, respectively. Clinical assessments and resting-state EEG recordings were conducted on Day 1 (baseline) and Day 5 before the stimulation session. After the treatment week, the group assignments were re-randomized, i.e., some participants initially in the tACS group crossed over to the sham group and vice versa. During the maintenance period, participants in the new tACS (sham) group received weekly clinical assessment and 10-Hz tACS (sham stimulation) for 40 min per visit. During 1-week, 1-month, and 2-month visits during the maintenance period, resting-state EEG of each participant was recorded after clinical assessments and before the stimulation session. Our analyses focused on the five key sessions where the resting-state EEG was recorded (S1-S5). The CONSORT flow diagram is shown in Fig. 2.

**Fig. 2:**
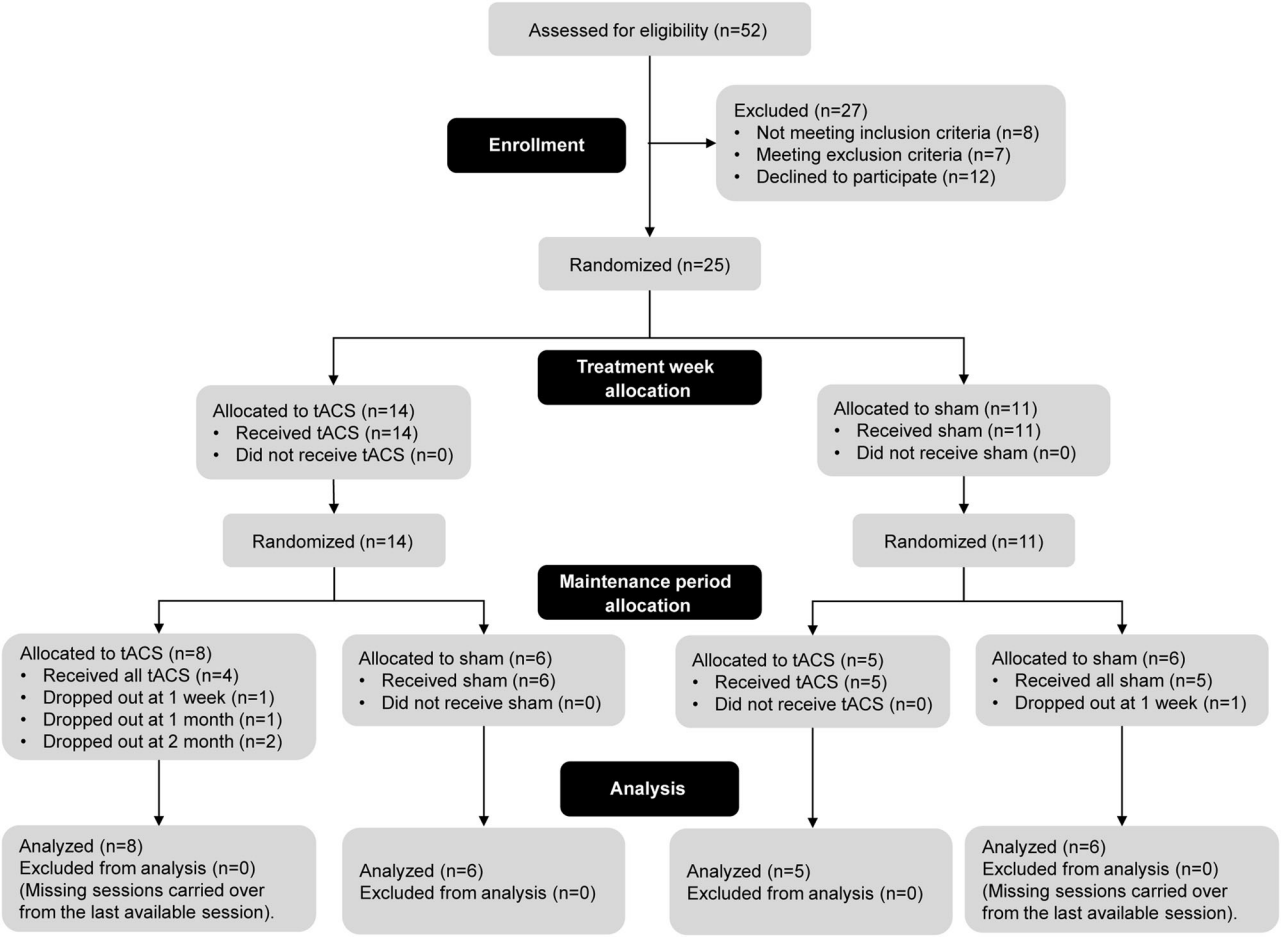
The CONSORT flow diagram.

**Fig. 3:**
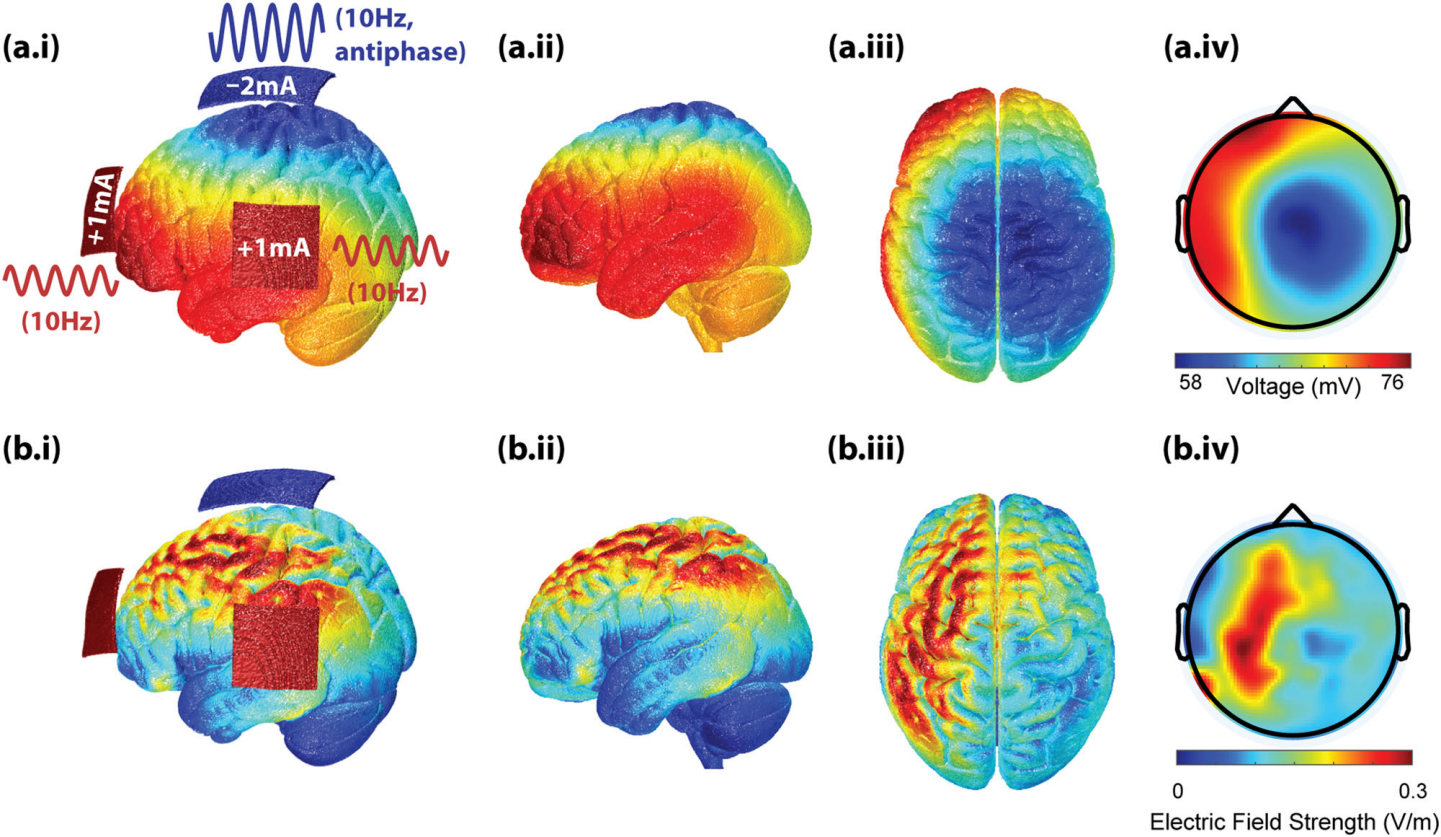
Transcranial alternating current stimulation (tACS) protocol. **a.i** alternating currents at 10 Hz were applied to the dorsolateral prefrontal cortex (dlPFC) and temporoparietal junction (TPJ) inphase with each other (red pads), which were antiphase with the return current at Cz (blue pad). tACS at PFC and TPJ has an amplitude of 1 mA (zero-to-peak), while the return current at Cz has an amplitude of 2 mA (zero-to-peak). (**a.i–a.iii**) show the simulated voltage distribution over the brain from three different views (left posterior, left, top) when currents at PFC and TPJ reach their positive peak. (**a.iv**) shows the topography of the simulated voltage distribution projected from the surface of the gray matter to the location of EEG electrodes. (**b.i-b.iii**) show the simulated electric field magnitude on the brain from three different views, and (b.iv) shows the projection of the electric field magnitude back to the level of EEG electrodes. (**a.iv**) and (**b.iv**) are shown here for direct visual comparisons with EEG results.

This study was a double-blind, randomized, placebo-controlled clinical trial with a cross-over design (Fig. 1; the full trial protocol can be found at clinicaltrials.gov). In the first stage of the study (the *treatment week*), participants were randomly assigned with equal probability to the tACS group or the placebo group: participants in the *tACS treatment group* received five consecutive days of 10 Hz tACS (Fig. 3a.i) for 40 mins each day, while participants in the placebo group (*sham treatment group*) received five consecutive days of sham stimulation for the same duration (see “Electrode montage and stimulation protocol” below for details). In the second stage of the study (the *maintenance period*), participants were re-assigned randomly with equal probability to the *tACS maintenance group* or the *sham maintenance group*. During this stage, participants in the tACS maintenance group received 10 Hz tACS for 40 mins during each of the weekly sessions (eight sessions in total from 1 week to 2 months after the treatment week), while participants in the sham maintenance group received sham stimulation during these sessions. Clinical assessments were conducted at the beginning of each session on Day 1 and Day 5 of the treatment week and for each weekly visit of the maintenance period (Fig. 1, clipboard symbols). Resting-state EEG recordings were conducted right after clinical assessments and right before the 40-min tACS or sham in selected sessions, namely, Day 1 and Day 5 of the treatment week and 1-week, 1-month, and 2-month follow-up of the maintenance period (labeled as S1–S5 in Fig. 1). See “Clinical outcome measures and statistics” and “Resting-state EEG” sections below for additional details. All randomization and group assignments were performed by a researcher otherwise unassociated with the study. The random sequences were generated by random permutations of group assignments (verum or sham) balanced in blocks of four. All researchers that participated in data collection and administering stimulation were blind to the group assignments. The blinded researchers were provided with a five-digit stimulation code to be entered into the stimulation device for each visit without knowing whether the code was associated with tACS or sham stimulation. The device recorded and encrypted the actual stimulation waveforms, which were decrypted and verified by the unblinded research personnel (Fig. 2).

### Electrode montage and stimulation protocol

Three carbon-silicone electrodes were applied to the scalp with Ten20 conductive paste (Bio-Medical Instruments, Clinton Township, MI) for all participants. One electrode of 5 × 5 cm was placed between F3 and Fp1 (in the International 10–20 system) in the area of dlPFC; another electrode of the same dimensions was placed between T3 and P3 in the area of the left TPJ. These two electrodes are shown as red pads in Fig. 3a.i, b.i. A third return electrode (5 × 7 cm) was placed over Cz (blue pad in Fig. 3a.i, b.i). For participants who received tACS, 10 Hz alternating currents were applied to the dlPFC and TPJ electrodes in phase to each other (red waves in Fig. 3a.i) with a zero-to-peak amplitude of 1 mA; 10-Hz alternating currents at the return electrode were antiphase to that of the dlPFC and TPJ electrodes (blue wave in Fig. 3a.i). The placement of electrodes was based on our previous work using tACS for the treatment of auditory hallucinations^49^ and earlier tDCS work^56,57^. The induced voltage distribution and the electric field strength shown in Fig. 3 were simulated using ROAST 3.0^58,59^ on the New York Head^60^. Participants in the tACS group received 10 Hz stimulation for 40 min. Participants in the sham group received 20 s tACS flanked by a 10 s ramp-up and a 10 s ramp-down to simulate the sensation of tACS on the skin for the purpose of blinding. During the stimulation session, participants were seated comfortably and presented with a video of tropical fish (Undersea Projections, Queensland, Australia) to minimize the perception of phosphenes that could be induced by stimulation.

### Clinical outcome measures and statistics

The primary outcome measure was the change in AHRS^61^ scores from baseline (Day 1) to the last day of the treatment week (Day 5) and to the last maintenance session (2-month follow-up). Secondary outcome measures included the change in Positive and Negative Syndrome Scale (PANSS)^5^ scores from Day 1 to Day 5 of the treatment week and to 2-month follow-up of the maintenance period. Other outcome measures the included change in Hamilton Program for Schizophrenia Voices Questionnaire (HPSVQ)^62^ scores from Day 1 to Day 5 of the treatment week and to the end of the 2-month maintenance period. For statistical analysis, we used a linear mixed-effect model analysis with fixed factors of “session” (S1–S5 in Fig. 1), “treatment week condition” (tACS vs. sham), and “maintenance period condition” (tACS vs. sham), and with “participant” as a random factor to account for repeated measures within participants. Kenward–Roger approximations were used to obtain *p*-values and perform F-tests for each factor and their interaction. Further analysis was performed using paired Student’s *t* tests to compare symptom severity on the last day of the treatment week or the last day of the maintenance period to that of baseline (Day 1); two-sample *t* tests were used to compare symptom changes in the tACS group to that of the sham group. Between-group comparisons in this study mainly focused on the contrast between those who received tACS vs. sham in the treatment week.

### Assessment of side effects and blinding

We administered an adverse effects questionnaire^46,51^ after each stimulation session. The questionnaire measured patient-reported headache, neck pain, scalp pain, tingling, itching, ringing/buzzing noise, burning sensation, local redness, sleepiness, trouble concentrating, improved mood, worsening of mood, dizziness, and flickering lights on a Likert scale from 1 (absent) to 4 (severe). To assess the effectiveness of the blinding procedures, after the final simulation session of the treatment week and the maintenance period, participants were asked whether they thought they had received tACS over the past period, and whether they thought their symptoms (auditory hallucinations) had improved. The study personnel was also asked whether they believed the participant had received tACS or sham during the treatment week and during the maintenance period.

### Resting-state EEG

Resting-state EEG data were sampled at 1000 Hz using a 128-channel Geodesic EEG system (EGI Inc., Eugene, OR) with Cz as the reference and an electrode between Cz and Pz as the ground. The session lasted 8 min for each participant, with interleaved eyes-open and eyes-closed blocks (2 min each), and the condition of the first block was randomized across participants. Eyes-open data were used to identify the individual alpha frequency and alpha power, and to calculate functional connectivity.

### EEG data analysis and statistics

#### Preprocessing

Data analyses were performed in Matlab using EEGLAB^63^ and FieldTrip^64^. All data were first down-sampled to 250 Hz with anti-aliasing filtering (lowpass at 0.9 of the Nyquist frequency, with transition bandwidth 0.2 of the Nyquist frequency). The down-sampled data were further bandpass filtered between 1 and 50 Hz. Artifact subspace reconstruction^65^ was used to automatically reject high amplitude artifacts and reconstruct signals from non-artifactual principle components. Bad channels are spatially interpolated. The data was then re-referenced to the common average. Further, infomax-independent component analysis (ICA)^66^ was used to remove eye blinks, eye movement, muscular artifacts, and heartbeats. All ICs were visually inspected, and artifactual components were manually selected for rejection. These preprocessing steps were carried out on the full dataset before unblinding the study.

#### Identify individual alpha frequency and power

Power spectral density was estimated using Welch’s method (in 2 s Hamming windows with 0.5 s overlap and 0.1 Hz spectral resolution). Aperiodic components were removed using FOOOF^67^ before extracting the individual alpha frequency (IAF; peak with the highest power density between 7 and 12 Hz, with consistent presence during the eyes-open blocks across all sessions). All spectra were visually inspected to confirm IAF choice. Alpha power was obtained by integrating the power density within ±1 Hz of the IAF for each channel and for each session. The topography of alpha power was visually inspected for each participant and session to check for any abnormality. One session of one participant was rejected (treated as missing data) due to abnormally high alpha power (600% greater than the baseline). All missing data were replaced with the value from the last available session.

#### Statistical comparisons of alpha power

We computed the percent signal change of alpha power for each session relative to the baseline session (Session 1 was a baseline for the treatment week, and after, Session 3 was a baseline for the maintenance period):

$${{{\mathrm{\% }}}}\;{\rm{change}}_n = \frac{{P_n - P_{{\rm{baseline}}}}}{{P_{{\rm{baseline}}}}}$$

where *P*_*n*_ is the alpha power in the *n*th session, and *P*_baseline_ is the alpha power in the baseline session. EEG channels with significant alpha power increase (% change > 0) were determined using a 1-sample cluster permutation test (right tail) to control for multiple comparisons (Mass Univariate ERP Toolbox^68^). In addition, we performed model-driven statistical analyses. We defined the region of interest (ROI) as the set of channels with the simulated electric field strength greater than 0.15 V/m (~half the maximum field strength). The average alpha power change was compared to zero (1-sample *t* tests) and across groups (2-sample *t* tests). The percent alpha power changes from baseline to Day 5 of the treatment week and to the 2-month follow-up are secondary outcome measures of the study in addition to PANSS. To test whether alpha power increase correlates topographically with electric field strength, we calculated the Pearson correlation coefficients between the topography of the simulated field strength (Fig. 3) and that of alpha power change.

#### Functional connectivity

Functional connectivity (phase synchrony) between any two-channel locations was measured by a de-biased weighted phase lag index (WPLI)^69^, which minimizes the effects of volume conduction and uncorrelated noise:

$${{\Phi }} = \frac{{\left| {E\left\{ {\left| {\Im \left\{ X \right\}} \right|{\rm{sign}}\left( {\Im \left\{ X \right\}} \right)} \right\}} \right|}}{{E\left\{ {\left| {\Im \left\{ X \right\}} \right|} \right\}}}$$

where $\Im \left\{ X \right\}$ denotes the imaginary part of the cross-spectrum and *E*{⋅} denotes the expectation. WPLI was computed for 1 to 20 Hz and 0.1 Hz resolution for all channel pairs on the scalp. To compute the global functional connectivity spectra^49^, WPLI at each frequency was further averaged across all scalp channel pairs. We extracted peak global functional connectivity frequency between 7 and 12 Hz. We compared peak frequency increase from baseline using 1-sample *t* tests (right tail). We also computed the average level of functional connectivity in specific regions of interest at the IAF or the stimulation frequency (10 Hz). The first region of interest was the target region of the stimulation, defined by the channels with a predicted electric field strength greater than 0.15 V/m (Figs. 3b.iv, 6c). Functional connectivity was averaged across all pairs of electrodes within the target region. In addition, we computed the average functional connectivity between the left and right frontal regions. The left frontal region was defined as F3 and its five surrounding electrodes, and the right frontal region was defined as F4 and its five surrounding electrodes (Fig. 7d). Frontal functional connectivity was included in the analysis due to its association with the severity of depressive symptoms^48^.

## Results

### Study sample

Twenty-five participants with clinically stable schizophrenia or schizoaffective disorder were randomly assigned to tACS or sham for the treatment week, during which they received 5 consecutive days of 10-Hz tACS or sham stimulation, respectively (Fig. 1, left). All participants completed the 5-day treatment period, after which participants were re-randomized to tACS or sham for the maintenance period, during which they received weekly 10-Hz tACS or sham stimulation in 8 visits (Fig. 1, right). Participants’ demographics, baseline symptom scores, and antipsychotic medication are reported in Table 1. There was no statistically significant difference in age or gender of participants across the four groups resulting from the two randomizations (Table 1, Demographics). Likewise, there was no statistically significant difference in baseline symptom measures across the four groups (Table 1, Baseline Symptoms). Participants who withdrew from the study during the maintenance period reported reasons unrelated to the stimulation. The study did not reach the target sample size of 40 due to recruitment difficulty, exacerbated by the COVID-19 pandemic. Thus, only 25 participants were recruited during the funded period.

**Table 1:** Participant demographics, baseline symptom scores, and antipsychotic medications by treatment week and maintenance period conditions.

	Treatment week + Maintenance period				
	tACS + tACS (*n* = 8)	tACS + Sham (*n* = 6)	Sham + tACS (*n* = 5)	Sham + Sham (*n* = 6)	Statistics
*Demographics*					
Age (Mean ± SD)	37 ± 13	40 ± 14	34 ± 11	45 ± 10	*F*_3,19_^a^ = 0.791 *p* = 0.514
Gender (Male)	5	4	3	4	*Χ*^2^ = 0.079 *p* = 0.994
*Baseline symptoms* (Mean ± SD)					
AHRS	25.0 ± 7.4	19.0 ± 6.8	23.4 ± 6.5	23.2 ± 7.0	*F*_3,21_ = 0.873 *p* = 0.471
HPSVQ	25.3 ± 9.1	24.8 ± 3.3	27.6 ± 3.2	25.2 ± 4.5	*F*_3,21_ = 0.231 *p* = 0.874
PANSS					
–Total	72.4 ± 19.4	59.0 ± 15.0	67.8 ± 10.1	66.0 ± 21.0	*F*_3,21_ = 0.685 *p* = 0.571
–Positive symptoms	19.5 ± 4.4	17.5 ± 4.9	18.0 ± 2.7	16.8 ± 5.4	*F*_3,21_ = 0.443 *p* = 0.725
–Negative symptoms	16.4 ± 6.3	13.8 ± 5.7	16.8 ± 4.2	18.2 ± 5.3	*F*_3,21_ = 0.630 *p* = 0.604
–General psychopathology	36.5 ± 11.5	27.7 ± 7.1	33.0 ± 5.0	31.0 ± 11.6	*F*_3,21_ = 1.02 p = 0.405
–Hallucinations	4.4 ± 0.7	4.8 ± 0.8	4.6 ± 1.1	4.8 ± 0.8	*F*_3,21_ = 0.484 p = 0.697
*Antipsychotic medication (n)*					
Aripiprazole	2	1	1	1	
Cariprazine	1				
Clozapine	3	3	1	1	
Fluphenazine		1			
Haloperidol	2		1		
Loxapine		1			
Lurasidone	1		1		
Olanzapine		2	1	2	
Paliperidone			1	1	
Perphenazine				2	
Quetiapine			1	1	
Risperidone				2	

Multiple participants used more than one type of antipsychotic medication.

^a^Two participants in the “tACS + Sham” group did not report their exact age.

### Effective blinding and low side effects

On the last day of the treatment week (Day 5) and that of the maintenance period (2-month follow-up), the participants were asked whether they believed they had received tACS treatment. For participants who received tACS in the treatment week, 9 out of 14 participants believed that they had received tACS. For participants who received sham stimulation, 8 out of 11 participants believed that they had received tACS instead of sham. For participants who received tACS in the maintenance period, 5 out of 10 participants believed that they had received tACS. For participants who received sham in the maintenance period, 8 out of 10 participants believed that they had received tACS instead of sham. Overall, there was no statistically significant difference between groups (Χ^2^(1) = 0.34 *p* = 0.56 for Day 5, and Χ^2^(1) = 0.64, *p* = 0.42 for 2-month follow-up). This suggests that the blinding was effective for the participants. In addition, the study personnel were asked whether they believed the participant had received tACS treatment. For the 14 participants who received tACS during the treatment week, the study personnel believed two of them had received tACS; for the 11 participants who received sham stimulation, the study personnel believed two of them had received tACS; there was no statistically significant difference between the two groups (Χ^2^(1)=0.034, *p* = 0.85). For the 10 participants who received tACS during the maintenance period, the study personnel believed one of them had received tACS; for the 10 participants who received sham during the maintenance period, the study personnel believed 3 of them had received tACS; there was no statistically significant difference between the two groups (Χ^2^(1)=0.44, *p* = 0.51). This suggests that the blinding was effective for the study personnel. Moreover, participants reported a low level of side effects (Table 2), mostly between 1 and 2 on a 1 to 4 scale. There was no statistical difference between the side effects between treatment groups. All participants completed all treatment week sessions, with five participants discontinued their participation during the maintenance period, 4 from the tACS treatment group and 1 from the sham treatment group. The reason for discontinuation of the one sham group participant was that the patient experienced an increase in psychotic symptom severity that is not unusual for the patient. The reasons for discontinuation of the four tACS group participants were: (1) the patient experienced an increase in psychotic symptom severity that is not unusual for the patient, (2) a family member believed that tACS was equivalent to electroconvulsive therapy and persuaded the patient to withdraw, (3) the patient was hospitalized for unrelated backpain that led to two missed sessions, and by study protocol, the patient was withdrawn by the study team, and (4) the patient was withdrawn by the study team per initial UNC School of Medicine COVID-19 policy. Thus, no evidence suggests that side effects had led to withdraws from the tACS group.

**Table 2:** Side effects by treatment week and maintenance period conditions.

	Treatment week + Maintenance period				
Side effect (Mean ± SD)	tACS + tACS (*n* = 8)	tACS + Sham (*n* = 6)	Sham + tACS (*n* = 5)	Sham + Sham (*n* = 6)	Statistics
Headache	1.05 ± 0.12	1.04 ± 0.07	1.02 ± 0.05	1.17 ± 0.28	*F*_3,21_ = 1.06 *p* = 0.387
Neck pain	1.13 ± 0.38	1.09 ± 0.19	1.08 ± 0.11	1.05 ± 0.09	*F*_3,21_ = 0.144 *p* = 0.932
Scalp pain	1.45 ± 0.51	1.33 ± 0.35	1.67 ± 1.13	1.36 ± 0.45	*F*_3,21_ = 0.309 *p* = 0.819
Tingling	2.02 ± 0.60	1.32 ± 0.35	1.95 ± 1.01	1.51 ± 0.29	*F*_3,21_ = 2.00 *p* = 0.144
Itching	1.49 ± 0.66	1.17 ± 0.30	1.33 ± 0.44	1.60 ± 0.62	*F*_3,21_ = 0.709 *p* = 0.557
Ringing/buzzing noise	1.13 ± 0.29	1.01 ± 0.03	1.01 ± 0.03	1.76 ± 0.97	*F*_3,21_ = 0.929 *p* = 0.444
Burning sensation	1.64 ± 0.74	1.71 ± 0.50	1.82 ± 1.02	1.76 ± 0.97	F_3,21_ = 0.053 p = 0.983
Local redness	1.02 ± 0.05	1 ± 0	1.15 ± 0.35	1 ± 0	*F*_3,21_ = 1.25 *p* = 0.317
Sleepiness	1.82 ± 1.00	1.41 ± 0.47	1.41 ± 0.35	1.41 ± 0.51	*F*_3,21_ = 0.624 *p* = 0.607
Trouble concentrating	1.32 ± 0.66	1.26 ± 0.35	1.10 ± 0.12	1.18 ± 0.38	*F*_3,21_ = 0.274 *p* = 0.843
Improved mood	1.89 ± 1.26	1.11 ± 0.26	1.22 ± 0.35	1.13 ± 0.28	*F*_3,21_ = 1.71 *p* = 0.195
Worsening of mood	1.14 ± 0.40	1 ± 0	1 ± 0	1.05 ± 0.13	*F*_3,21_ = 0.541 *p* = 0.659
Dizziness	1.08 ± 0.19	1.08 ± 0.20	1.01 ± 0.03	1.05 ± 0.06	*F*_3,21_ = 0.243 *p* = 0.865
Flickering Lights	1.05 ± 0.83	1.70 ± 0.61	1.31 ± 0.51	1.25 ± 0.49	*F*_3,21_ = 0.578 *p* = 0.636

Side effects questionnaires were administered after each stimulation session, during which the participant either received tACS or sham stimulation. Each participant’s side effect scores were averaged across all sessions for group comparisons (F-statistics).

### Clinical outcomes

Group-averaged symptom scores in two key visits, the last day of the treatment week (Day 5) and the last day of the maintenance period (2-month follow-up), are shown in Table 3. First, we used Kenward–Roger’s F-tests to estimate the overall statistical significance of the effect of tACS on symptom severity across all sessions, which was captured by the interaction between group assignments and sessions in linear mixed-effect models. No significant interaction was found between treatment week or maintenance week condition and symptom changes across sessions (Table 4), meaning the overall trajectories of symptom changes were not significantly different between tACS and sham groups. Note that the residuals of the linear models were not always normally distributed or homoscedastic (see Supplementary Table S1 for diagnostic statistics). Thus, the p-values in Table 4 should be interpreted with caution. The lack of significant interaction effects may reflect insufficient sensitivity of the test to non-normally distributed data. There was a significant main effect of the session on HPSVQ and PANSS scores, which reflect an overall decreasing trend of symptoms over time. There was a significant main effect of maintenance week condition on AHRS scores, meaning there was a difference between the overall auditory hallucination severity between people assigned to receive tACS vs. sham during the maintenance period.

**Table 3:** Symptoms scores on the last day of the treatment week (Day 5) and the last day of the maintenance period (2-month follow-up).

	Treatment week + Maintenance period			
Symptom scores (Mean ± SD)	tACS + tACS (*n* = 8)	tACS + Sham (*n* = 6)	Sham + tACS (*n* = 5)	Sham + Sham (*n* = 6)
*Day 5*				
AHRS	25.1 ± 8.0	19.8 ± 5.1	25.6 ± 6.8	21.0 ± 5.9
HPSVQ	25.9 ± 8.4	23.2 ± 5.3	26.4 ± 5.7	23.2 ± 1.8
PANSS				
–Total	66.1 ± 14.7	58.2 ± 14.6	67.8 ± 5.0	64.7 ± 17.9
–Positive symptoms	18.6 ± 4.0	17.2 ± 4.7	16.6 ± 3.4	17.3 ± 5.2
–Negative symptoms	15.5 ± 5.1	15.0 ± 7.0	17.6 ± 6.5	17.7 ± 3.5
–General psychopathology	32.0 ± 8.4	26.0 ± 5.7	22.6 ± 2.4	29.7 ± 10.3
–Hallucinations	4.4 ± 0.7	4.8 ± 0.8	4.6 ± 0.5	4.7 ± 1.0
*2-month follow-up*				
AHRS	23.9 ± 9.6	15.7 ± 5.0	26.4 ± 6.8	17.6 ± 7.1
HPSVQ	25.6 ± 9.8	20.5 ± 5.7	26.5 ± 4.2	20.3 ± 4.6
PANSS				
–Total	67.0 ± 10.1	58.0 ± 12.6	65.2 ± 5.2	60.0 ± 20.4
–Positive symptoms	18.3 ± 3.9	17.0 ± 3.2	17.2 ± 2.17	15.7 ± 5.2
–Negative symptoms	16.3 ± 5.3	15.7 ± 6.8	16.8 ± 4.4	15.5 ± 5.0
–General psychopathology	32.5 ± 5.8	25.3 ± 5.4	31.2 ± 2.5	28.8 ± 11.0
–Hallucinations	4.4 ± 0.7	4.3 ± 0.8	4.6 ± 0.5	4.5 ± 0.8

**Table 4:** F-test results of treatment and maintenance effects using Kenward-Roger Approximation.

	Main effects			Interactions	
	Treatment	Maintenance	Session	Treatment x Session	Maintenance x Session
AHRS	*F*_1,22_ = 0.053	*F*_1,22_ = 4.73	*F*_4,96_ = 1.56	*F*_4,88_ = 1.26	*F*_4,88_ = 2.29
	*p* = 0.820	***p*** **= 0.041**	*p* = 0.190	*p* = 0.292	*p* = 0.066
HPSVQ	*F*_1,22_ = 0.045	*F*_1,22_ = 1.98	*F*_4,96_ = 3.17	*F*_4,88_ = 0.179	*F*_4,88_ = 2.24
	*p* = 0.834	*p* = 0.174	***p*** **= 0.017**	*p* = 0.949	*p* = 0.070
PANSS					
–total	*F*_1,22_ = 0.093	*F*_1,22_ = 1.54	*F*_2,48_ = 3.58	*F*_2,44_ = 0.911	*F*_2,44_ = 0.326
	*p* = 0.764	*p* = 0.227	***p*** **= 0.036**	*p* = 0.409	*p* = 0.724
–Positive symptoms	*F*_1,22_ = 0.455	*F*_1,22_ = 0.542	*F*_2,48_ = 1.86	*F*_2,44_ = 0.029	*F*_2,44_ = 0.676
	*p* = 0.507	*p* = 0.470	*p* = 0.167	*p* = 0.972	*p* = 0.514
–Negative symptoms	*F*_1,22_ = 0.611	*F*_1,22_ = 0.098	*F*_2,48_ = 0.130	*F*_2,44_ = 2.21	*F*_2,44_ = 0.177
	*p* = 0.443	*p* = 0.758	*p* = 0.879	*p* = 0.122	*p* = 0.838
–General psychopathology	*F*_1,22_ = 0.147	*F*_1,22_ = 2.98	*F*_2,48_ = 5.28	*F*_2,44_ = 1.18	*F*_2,44_ = 0.117
	*p* = 0.705	*p* = 0.098	***p*** **= 0.008**	*p* = 0.316	*p* = 0.890
–Hallucinations	*F*_1,22_ = 0.150	*F*_1,22_ = 0.430	*F*_2,48_ = 2.20	*F*_2,44_ = 0.334	*F*_2,44_ = 2.59
	*p* = 0.703	*p* = 0.519	*p* = 0.122	*p* = 0.718	*p* = 0.086

A significant interaction between Treatment or Maintenance condition with Session would indicate a significant difference between tACS and sham group over time. No statistically significant interaction effects were observed.

**Fig. 4:**
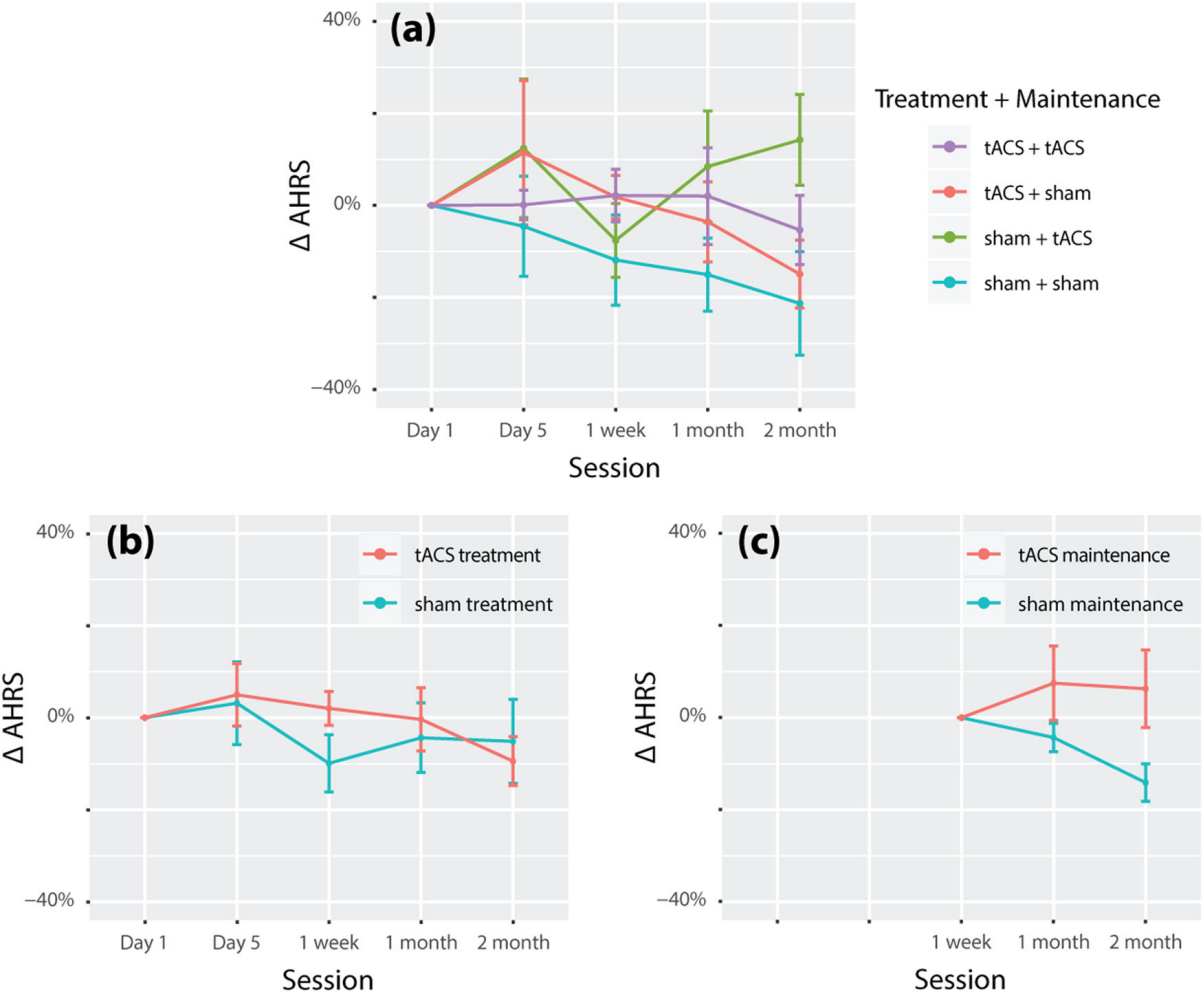
No significant change in auditory hallucinations from baseline following tACS treatment or maintenance. **a** The percent symptom changes in the auditory hallucination rating scale (AHRS) from baseline (Day 1) in four groups by whether the participants had received 10 Hz-tACS or sham stimulation during the treatment week and the maintenance period. **b** The percent symptom changes in AHRS from Day 1 in two groups by whether the participants had received 10 Hz-tACS or sham stimulation during the treatment week. **c** The percent symptom changes in AHRS from 1-week follow-up in two groups by whether the participants had received 10 Hz-tACS or sham stimulation during the maintenance period.

Next, we examine the changes in the primary outcome measure of auditory hallucinations, AHRS, from the baseline to specific sessions (Fig. 4). There was no substantial change in AHRS from Day 1 to Day 5 for both the tACS and the sham treatment groups (Cohen’s d < 0.2, Table 5; 95% upper bounds are 2.25 and 3.51, respectively; Fig. 4b). At 2-month follow-up, the tACS treatment group did show a marginally significant decrease in AHRS with an effect size of 0.450 (*p* < 0.01, 95% upper bound = 0.10) comparable to our previous study^49^. The sham treatment group also showed a numerical decrease in AHRS, with an effect size of 0.239 (non-significant, 95% upper bound = 2.17). Compared to the maintenance period baseline (week 1, Fig. 4c), the tACS maintenance group showed negligible change in AHRS (t(12) = 0.539, *p* = 0.700, d = 0.149; 95% upper bound = 3.48) while the sham group showed a significant decrease (t(11) = −3.37, *p* = 0.003, *d* = −0.973; 95% upper bound = −1.23). Overall, there was no session-specific treatment effect of tACS, either during the treatment week or the maintenance period, on auditory hallucination symptoms.

**Table 5:** The statistical comparisons and effect sizes of symptom reduction from baseline.

	Day 5		2-month follow-up	
Outcome\treatment	tACS	Sham	tACS	Sham
AHRS	*t*_13_ = 0.416	*t*_10_ = −0.089	*t*_13_ = −1.68	*t*_10_ = −0.792
	*p* = 0.658	*p* = 0.465	*p* = 0.058^†^	*p* = 0.223
	*d* = 0.111	*d* = −0.026	i = −0.450	i = −0.239
HPSVQ	*t*_13_ = −0.28	i_10_ = −1.69	*t*_13_ = −1.16	*t*_10_ = −2.08
	*p* = 0.390	*p* = 0.060^†^	*p* = 0.134	***p*** = 0.032*
	*d* = −0.076	*d* = −0.511	*d* = −0.309	***d*** **= −0.628**
PANSS				
–total	*t*_13_ = −1.87	*t*_10_ = −0.451	*t*_13_ = −1.18	*t*_10_ = −2.43
	***p*** = 0.042*	*p* = 0.331	*p* = 0.130	***p*** = 0.018*
	***d*** **= −0.499**	*d* = −0.136	*d* = −0.314	***d*** **= −0.734**
–Positive symptoms	*t*_13_ = −1.06	*t*_10_ = −0.396	*t*_13_ = −1.11	*t*_10_ = −2.14
	*p* = 0.155	*p* = 0.350	*p* = 0.143	***p*** = 0.029*
	*d* = −0.283	*d* = −0.120	*d* = −0.297	***d*** **= −0.645**
–Negative symptoms	*t*_13_ = 0	*t*_10_ = 0.1	*t*_13_ = 0.764	*t*_10_ = −1.69
	*p* = 0.5	*p* = 0.539	*p* = 0.771	*p* = 0.060^†^
	*d* = 0	*d* = 0.030	*d* = 0.204	*d* = −0.512
–General psychopathology	*t*_13_ = −2.58	*t*_10_ = −0.470	*t*_13_ = −1.98	*t*_10_ = −1.87
	***p*** = 0.011*	*p* = 0.324	***p*** = 0.034*	***p*** = 0.046*
	***d*** **= −0.690**	*d* = −0.142	***d*** **= −0.530**	***d*** **= −0.563**
–Hallucinations	*t*_13_ = 0	*t*_10_ = −0.559	*t*_13_ = −1.38	*t*_10_ = −1
	*p* = 0.5	*p* = 0.294	*p* = 0.095^†^	*p* = 0.170
	*d* = 0	*d* = −0.169	*d* = −0.370	*d* = −0.302

Statistical results shown are based on paired *t* tests of the decrease of symptom scores from baseline at the end of the treatment week (Day 5) and the end of the maintenance period (2-month follow-up) for participants who received 5-day tACS or sham stimulation during the treatment week. Bold: statistically significant results. *P*-values were computed using left-tail paired *t* tests. (*p < 0.05. ^†^*p* < 0.10).

**Fig. 5:**
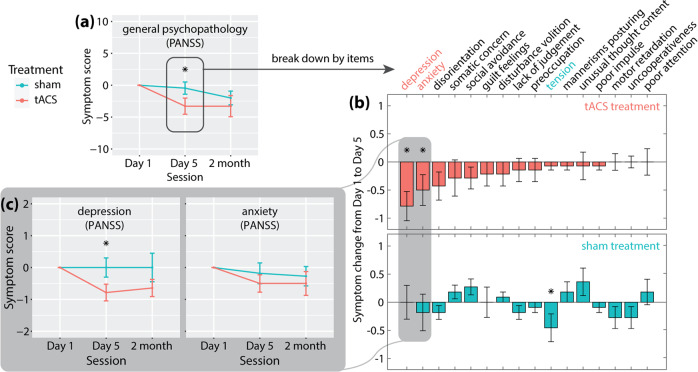
Breaking down the change in general psychopathology. **a** the tACS treatment group, but not the sham group, exhibited a significant decrease in PANSS general psychopathology subscore from Day 1 (baseline) to Day 5 (end of the treatment week). Breaking down the subscore to individual items (**b**), we observed an across-the-board decrease in symptom scores for the tACS treatment group. The patterns of symptom change were distinct between tACS and sham treatment groups (see text for statistical details). There was a significant decrease in depression and anxiety scores from baseline to Day 5 in the tACS treatment group but not in the sham group. The change in depression score from baseline to Day 5 was significantly greater in the tACS treatment group than of the sham group (**c**, left). The change in anxiety scores was not significantly different between tACS and sham treatment groups.

Further, we examined the symptom change from baseline to Day 5 and 2-month follow-up for secondary outcome measures by the treatment week condition (Table 5). For the tACS treatment group, a significant symptom reduction was observed for PANSS general psychopathology subscore on both Day 5 of the treatment week (effect size −0.690, *p* < 0.05, 95% upper bound = −1.03), and at two-month follow-up (effect size −0.530, *p* < 0.05, 95% upper bound = −0.35). The baseline-to-Day 5 reduction of PANSS general psychopathology subscore in the tACS treatment group was significantly greater than that of the sham group (*p* < 0.05, Fig. 5a, 95% upper bound = −0.09). In contrast, the tACS maintenance group showed virtually no change across all symptom measures (*p* > 0.05, not shown).

As a further exploratory analysis, we broke down the change of PANSS general psychopathology subscore from Day 1 to Day 5 into individual items (Fig. 5b). In the tACS treatment group, there was an across-the-board decrease in symptoms for almost all PANSS general psychopathology subscale items (Fig. 5b, upper panel). In particular, depression and anxiety scores significantly decreased from Day 1 to Day 5 of the treatment week (depression: −0.79 ± 0.97, t (13) = −3.02, *p* < 0.005, Cohen’s d = −0.806; anxiety: −0.50 ± 1.02, t (13) = −1.84, *p* < 0.05, Cohen’s d = −0.491; highlighted in Fig. 5b). In contrast, the sham treatment group exhibited a significant decrease in the tension score but not for depression or anxiety. The decrease in depression score on Day 5 was significantly greater in the tACS treatment group than in the sham group (*p* < 0.05, Fig. 5c, left). The decrease in anxiety score was not significantly different between the two groups (*p* = 0.23, Fig. 5c, right). Overall, the symptom changes in the tACS vs. sham treatment group followed distinct patterns on Day 5 of the treatment week (Pearson correlation between the group-average symptom scores of the tACS group and that of the sham group across all items in general psychopathology subscale: *r* = −0.048, *p* = 0.86; Fig. 5b) and at 2-month follow-up (*r*= −0.22, *p* = 0.41). As a step of further validation, we used a consensus five-factor model of PANSS^70^ to examine whether the decrease of depression symptoms in the tACS treatment group remain significant after correction for false discovery rate (FDR) of multiple comparisons. The five factors of the model are Positive factor, Negative factor, Disorganized/concrete factor, Excited factor, and Depressed factor. Consistently with above item-level findings, we found that the Depressed factor significantly decreased from baseline to Day-5 of the treatment week in the tACS treatment group (*p*_FDR_ = 0.0295) but not in the sham treatment group (*p*_FDR_ = 1). Other factors exhibited no significant changes in either group (*p*_FDR_ = 1).

### tACS treatment increases alpha power and alters functional connectivity

**Fig. 6:**
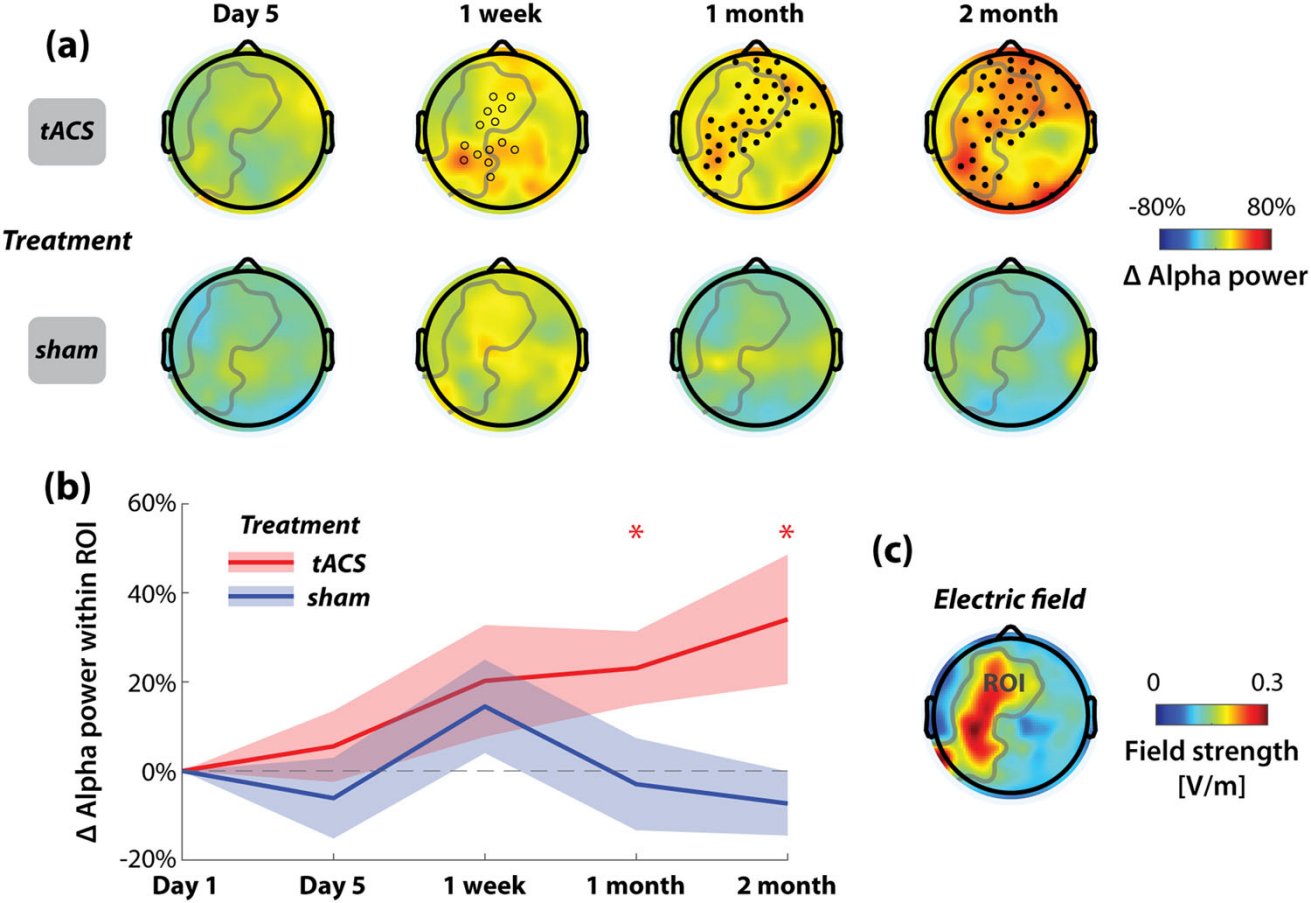
Alpha power increased over two months for the tACS treatment group. (**a**) shows the group average of alpha power change from the baseline (Day 1) to four main follow-up visits by the treatment condition (top row: 10Hz-tACS, bottom row: sham). Solid dots mark the clusters with a statistically significant increase in alpha power (*p* < 0.05); open circles mark the clusters with a marginally significant increase in alpha power (*p* < 0.1). Only the tACS group exhibited significantly increased alpha power from baseline. Area enclosed by the gray curve is the target region, a predefined region of interest (ROI) based on the simulated electric field (**c**) with field strength greater than 0.15 V/m. (**b**) shows the average alpha power change (mean ± standard error shown as solid lines and shaded areas) within the target region for the tACS treatment group and the sham treatment group. (*p < 0.05 significant increase from baseline for the tACS group, FDR-corrected).

We found a significant increase in alpha power from the baseline (Day 1) in participants in the tACS treatment group but not for participants in the sham treatment group (Fig. 6a, b). Interestingly, a significant power increase was not observed on the last day of the treatment week (Day 5, Fig. 6a top row, *p* > 0.1 for cluster permutation test over all scalp electrodes) as we had predicted based on our previous work^49^. Rather, alpha power continued to increase over the 2-month maintenance period, reaching statistical significance (*p* < 0.05, cluster permutation test) at 1-month and 2-month follow-up (Fig. 6a, top row, right two plots). Electrodes with significant alpha power increase largely overlapped with the target region (gray curve enclosed area in Fig. 6a top row). The target region (ROI in Fig. 6c) was defined using the simulated electric field where the field strength was greater than 0.15 V/m. Average alpha power change within the target region for the tACS group increased over time (Fig. 6b) and reached significance at 1-month and 2-month follow-ups. Moreover, we found that the group-average topography of alpha power increase in the tACS group (Fig. 6a, top row) was significantly correlated with the topography of the simulated electric field at 1-month follow-up (*r* = 0.23, *p* = 0.015, right tail) and 2-month follow-up (*r* = 0.21, *p* = 0.024, right tail). No significant alpha power increase was observed for the sham group (Fig. 6a, bottom row, and b). In contrast to the effect of treatment tACS, no significant alpha power increase was observed for participants who received weekly maintenance tACS (*p* > 0.1 for cluster permutation tests over all scalp electrodes; not shown).

**Fig. 7:**
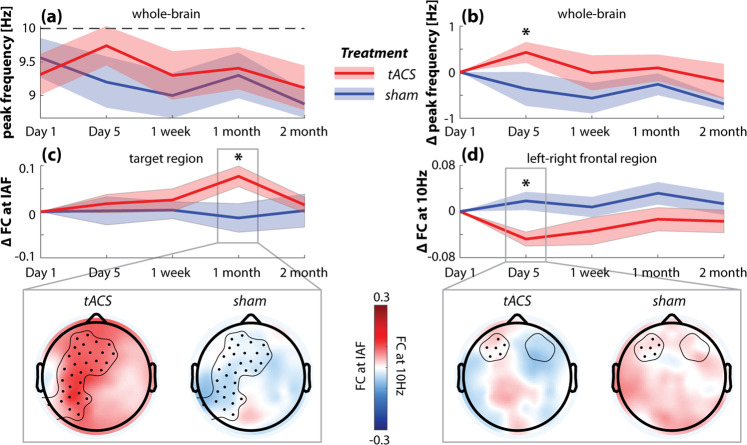
Functional connectivity was altered in frequency and in level for the tACS treatment group. Functional connectivity (FC) between any two channels was computed as the weighted phase lag index (WPLI) between the wavelet transform of the signals at each frequency (0.1 Hz resolution). **a** Peak frequency of whole-brain FC was extracted from 7 to 12 Hz for each subject and averaged for tACS and sham treatment groups separately (solid line = mean, colored band = standard error). **b** As expected based on a previous study^49^, the tACS treatment group, which received 10 Hz tACS in the treatment week, showed a significant increase of peak connectivity frequency towards 10 Hz on Day 5. This FC peak frequency change was significantly greater in the tACS treatment group than in the sham group. (**c**) shows the change in FC within the target region (electric fields strength > 0.15 V/m) at the individual alpha frequency (IAF). There was a significant increase of FC in the target region at 1-month follow-up for the tACS treatment group compared to the sham group. The topographic plots show the average FC from each electrode to all electrodes in the target region (dots) for the two groups. (**d**) shows the change in FC between the left and right frontal regions, defined by F3 and F4 with 5 surrounding electrodes for each (circled). Frontal FC significantly decreased from baseline on Day 5 in the tACS treatment group compared to the sham group. The topographic plots show the FC from each electrode to the left frontal region of interest (electrodes shown as dots). (*p < 0.05).

To examine how 10 Hz-tACS affected functional connectivity between brain regions, we computed WPLI for each pair of scalp electrodes at each frequency (7–12 Hz at 0.1 Hz resolution). Global (whole brain) functional connectivity at each frequency was defined by the average WPLI over all scalp electrode pairs. We extracted the frequency of peak global functional connectivity between 7 and 12 Hz for each participant and each session. Figure 7a shows the average frequency of peak functional connectivity for two groups—participants who received 10 Hz-tACS (red) versus those who received sham stimulation (blue) during the treatment week. On the last day of the treatment week (Day 5, Fig. 7a), peak frequency for the tACS group increased towards 10 Hz (dashed line), while that of the sham group decreased slightly. Statistically, we calculated peak frequency change relative to the baseline (Day 1, Fig. 7b) and found that peak frequency on Day 5 for the tACS group had significantly increased from baseline (*t*(13) = 1.90, *p* = 0.040), which is significantly higher than that of the sham group (*t*(23) = 1.92, *p* = 0.034). This result replicates our previous finding of increasing peak functional connectivity frequency towards 10 Hz in participants who received 10 Hz tACS treatment^49^. We also compared peak frequency change from baseline (1-week follow-up) by the maintenance period condition (not shown), but no statistically significant change was observed.

In addition to the peak frequency change, we examined functional connectivity at the IAF or the stimulation frequency 10 Hz (averaged within ± 1 Hz) within specific regions of interest (Fig. 7c, d). First, we found functional connectivity within the target region (c.f. Fig. 6c) significantly increased from baseline in the tACS treatment group compared to the sham group at 1-month follow-up (*t*(23) = 2.43, *p* = 0.01; Fig. 7c). For exploratory analysis, we examined functional connectivity between the left and right frontal regions at the stimulation frequency, which has been previously associated with the severity of depressive symptoms^48^. We found a significant reduction of frontal functional connectivity on Day 5 of the treatment week in the tACS treatment group compared to the sham group (*t*(23) = −3.37, *p* = 0.0013; Fig. 7d).

To ensure that the EEG results are not the mere consequence of differential signal quality in different groups, we performed additional statistical analysis to compare the number of components rejected during preprocessing. The number of components rejected for participants in the tACS treatment group was 44 ± 15 (range 11–73), and that of the sham treatment group was 43 ± 17 (range 8–91). There was no significant difference between the tACS treatment group and sham treatment group (aggregated over all sessions: *t*(123) = 0.57, *p* = 0.57; for each single session: *p* > 0.3). Similarly, the number of components rejected for participants in the tACS maintenance group was 45 ± 15 (range 8–91), and that of the sham maintenance group was 42 ± 14 (range 11–78). There was no significant difference between the tACS maintenance group and sham maintenance group (aggregated over all sessions: *t*(123) = 1.29, *p* = 0.2; for each single session: *p* > 0.09). Thus, EEG findings above cannot be explained by intergroup difference in signal quality.

### Changes in functional connectivity are associated with changes in general psychopathology and depression symptoms

**Fig. 8:**
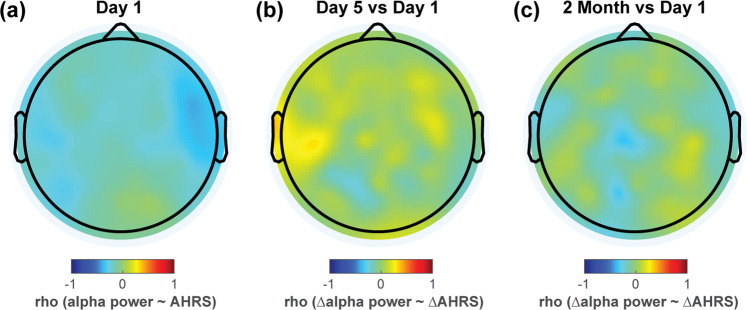
No significant correlation between alpha power and auditory hallucinations. (**a**) shows the topography of the correlation coefficient (rho) between alpha power and AHRS scores across all participants at baseline (Day 1). No significant correlation was observed for scalp electrodes. Change from baseline to the end of the treatment week (Day 5) was not significantly correlated with changes in AHRS (**b**). Likewise, the change from baseline to the final day of the maintenance period (2-month) was not significantly correlated with AHRS changes (**c**). (all *p* > 0.1 with FDR correction).

First, we examined the relationship between the primary EEG and clinical outcomes, i.e., the alpha power and the auditory hallucinations as measured by AHRS. At baseline (Day 1, Fig. 8a), we did not find a statistically significant correlation between alpha power and AHRS for any scalp electrodes (*p* > 0.1 with FDR correction, including all participants). Further, we did not find a statistically significant correlation between change of alpha power and change of AHRS from baseline (Day 1) to the final day of the treatment week (Day 5, Fig. 8b) or to the final day of the maintenance period (2-month follow-up, Fig. 8c; *p* > 0.1 with FDR correction). Likewise, no significant correlation was observed between the change in alpha power and change in symptom severity from baseline of the maintenance period (1-week follow-up) to the last day of the maintenance period (2-month follow-up; *p* > 0.1 with FDR correction, not shown).

In exploratory analyses, we examined the correlation between alpha power within the target region (ROI, Fig. 6c) and other symptom measures. At baseline, alpha power within the target region was not significantly correlated with symptom severity as measured by HPSVQ, PANSS, or PANSS general psychopathology subscore (*p* > 0.2, two-tail, Spearman correlation across all participants). The change in alpha power in the target region from baseline to 2-month follow-up was positively correlated with PANSS general psychopathology subscore (Spearman correlation, rho = 0.458, *p* =0.02, two-tail). Further analysis showed that this positive correlation was present only in the sham treatment group (rho = 0.68, *p* = 0.022) but not in the tACS treatment group (rho = 0.42, *p* = 0.14). Changes in other symptom measures were not correlated with alpha power change within the target region.

**Fig. 9:**
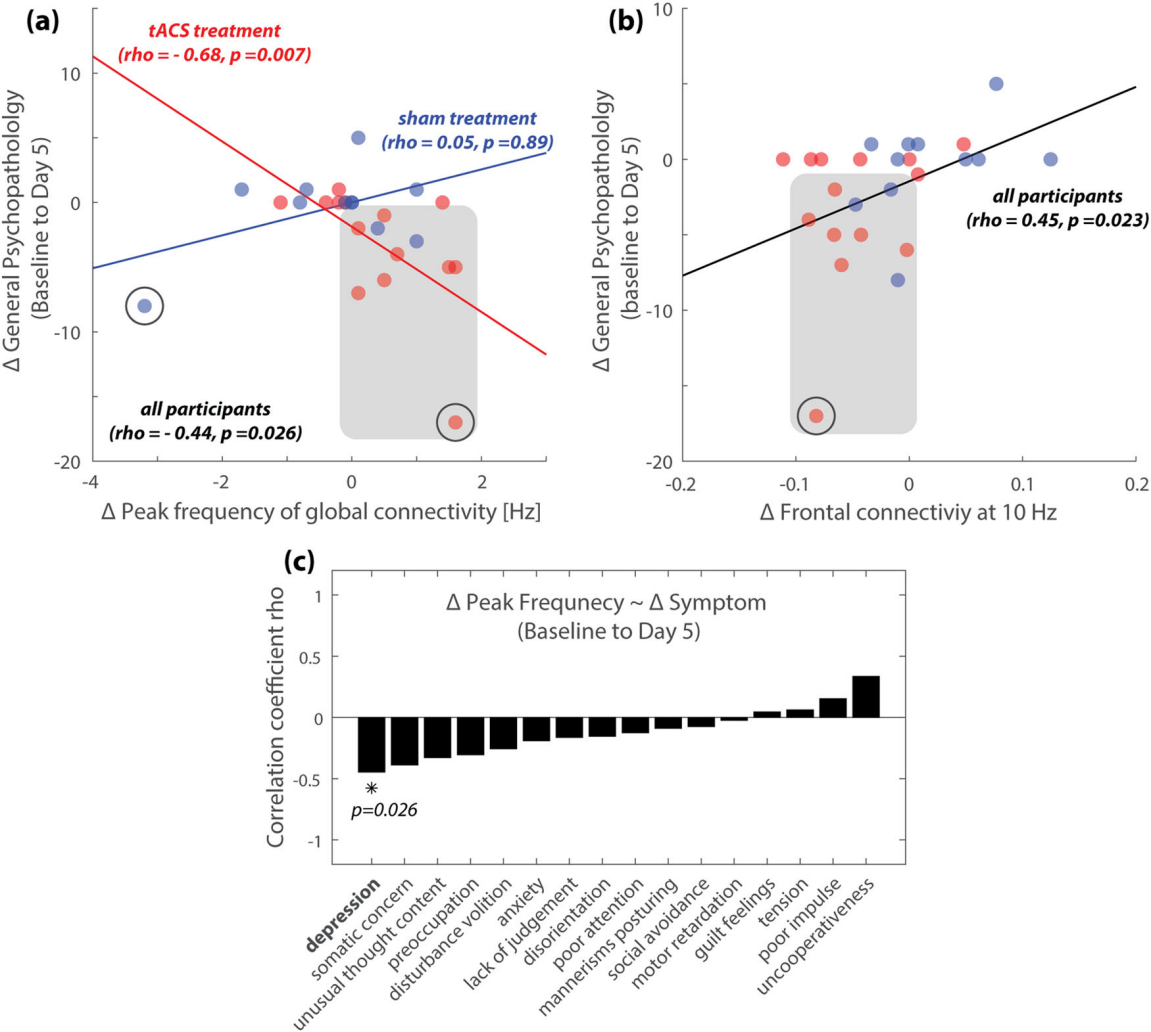
Changes in functional connectivity correlated with changes in general psychopathology and depressive symptoms. **a** The change in the peak frequency of global functional connectivity was negatively correlated with the symptom severity in PANSS general psychopathology across all participants and within the tACS treatment group. 8 of 14 participants in the tACS treatment group exhibited a decrease in general psychopathology. Remarkably, 100% of these participants also exhibited an increase in peak frequency of global functional connectivity (red dots in shaded area). Conversely, 9 out of 14 participants in the tACS treatment group exhibited an increase in peak frequency, and 8 of 9 of those participants exhibited a decrease in general psychopathology (with one participant with no change in symptoms). A further breakdown of PANSS general psychopathology subscore showed a significant correlation between peak frequency change and depression (**c**). **b** Changes in frontal functional connectivity at the stimulation frequency (10 Hz) were positively correlated with that of PANSS general psychopathology subscore. In other words, a decrease in frontal connectivity improved symptoms. Among the 8 out of 14 participants in the tACS treatment group who exhibited a decrease in symptom severity on Day 5, seven of them also exhibited a decrease in frontal connectivity (shaded area; one participant had virtually no change in frontal connectivity). Note that, in (**a**) and (**b**), functional connectivity changes and symptom changes are shown in the original scale for visualization. The statistical results are based on Spearman correlation. Spearman correlation was used for its robustness against outliers (circled). Removing the circled sample points led to qualitatively equivalent results (see text for details).

Next, we examined the relationship between peak frequency of global functional connectivity and auditory hallucinations as well as other symptom measures. At baseline, peak frequency was not correlated with auditory hallucination symptoms as measured by AHRS (rho = 0.063, *p* = 0.77) or HPSVQ (rho = 0.048, *p* = 0.82). Baseline peak frequency was also not correlated with other symptom measures (PANSS total score, PANSS general psychopathology subscore, or PANSS depression score, *p* > 0.4). Changes in peak frequency of global functional connectivity (c.f. Fig. 7b) were not significantly correlated with changes in auditory hallucinations as measured by AHRS or HPSVQ from baseline to Day 5 (AHRS: rho = 0.10, *p* = 0.64; HPSVQ: rho = −0.11, *p* = 0.59) or to 2-month follow-up (AHRS: rho = 0.009, *p* = 0.97; HPSVQ: rho = 0.29, *p* = 0.15). On the other hand, the change in peak frequency from baseline to Day 5 was negatively correlated with PANSS general psychopathology subscore (Fig. 9a; with all participants included, Spearman’s rho = −0.44, *p* = 0.026; with circled participants excluded, rho = −0.57, *p* < 0.005). This negative correlation was pronounced in the tACS treatment group (red in Fig. 9a; with all participants included, rho = −0.68, *p* = 0.007; with circled participants excluded, rho = −0.61, *p* = 0.027), but not significant in the sham group (blue in Fig. 9a; with all participants included, rho = 0.05, *p* = 0.89; with circled participants excluded, rho = −0.29, *p* = 0.422). A further breakdown of PANSS general psychopathology into individual items revealed a significant negative correlation between peak frequency change and change in depression (Fig. 9c). No significant correlation was found between change in peak frequency and change in symptom measures from baseline to 2-month follow-up (*p* > 0.05).

We also examined the relationship between the level of left-right frontal connectivity at 10 Hz and PANSS general psychopathology symptoms. At baseline, we did not observe a significant correlation between frontal connectivity and PANSS general psychopathology subscore or the depression score. On the other hand, change in frontal connectivity from baseline to Day 5 of the treatment week (Fig. 7d) was positively correlated with PANSS general psychopathology subscore, meaning decreasing frontal connectivity on Day 5 improved symptoms (Fig. 9b; with all participants included, rho = 0.45, *p* = 0.023; with circled participant removed, rho = 0.41, *p* = 0.045). The correlation between changes in the depression score and changes in frontal connectivity was positive but not statistically significant (rho = 0.26, *p* = 0.21, two-tail). We also examined the relationship between changes in functional connectivity within the target region (Fig. 7c) and changes in symptoms from baseline to 1-month follow-up and did not observe any statistically significant correlations.

## Discussion

The present study examined the effect of repeated 10-Hz tACS on brain dynamics and symptom presentation of people with schizophrenia and auditory hallucinations. At the neural level, tACS treatment successfully engaged the treatment target by increasing alpha power and increasing the peak frequency of global functional connectivity towards 10 Hz, in close agreement with the findings of our previous double-blind clinical trial^49,51^. At the symptom level, tACS treatment reduced general psychopathology and depression symptoms but not auditory hallucinations when compared to placebo. Importantly, the increase of peak frequency of brain functional connectivity was significantly correlated with a reduction of symptom severity in general psychopathology and depression. The work demonstrates reproducible target engagement by tACS in people with schizophrenia and auditory hallucinations and provides initial evidence for the use of tACS as a transdiagnostic treatment for depressive symptoms.

In the present study, daily alpha tACS induced changes in both alpha power and alpha functional connectivity but over different time scales. Alpha power gradually increased over the course of two months after the initial 5-Day stimulation, while changes in functional connectivity at or toward the stimulation frequency 10 Hz were immediate. Accompanying this contrast in time scales is that only changes in functional connectivity were related to symptom improvement while changes in alpha power were not. These observations suggest that changes in alpha power and alpha functional connectivity reflect distinct neural dynamical processes and functional roles in cognition.

The gradual, rather than immediate, increase of alpha power suggests that initial repeated stimulation may have triggered continuous network changes due to neuronal plasticity. Endogenous brain oscillations are thought to facilitate synaptic plasticity by bringing neuronal spikes to close temporal alignment, which may further reinforce the macroscopic oscillation^71,72^. Synaptic plasticity is considered an explanation for the lasting after-effect of alpha-tACS on endogenous oscillations^73,74^. This is particularly important in the context of the treatment of psychiatric disorders since it is desirable to induce long-lasting effects with limited stimulation duration. However, we are far from understanding what the optimal stimulation protocol is for multiple-session, multiple-day tACS to induce long-term alpha power increase. For example, weekly tACS during the maintenance period in the present study did not increase alpha power even though total stimulation time and thus cumulative dose was comparable to the 5-Day stimulation, suggesting that the multiple stimulation sessions need to be sufficiently close in time (e.g., daily) to trigger the presumed plasticity. The long-term dynamics of alpha oscillations may also be state-dependent and sensitive to individual variability. In our previous study^49^, alpha power increased the most on Day 5 and slightly diminished at 1-week and 1-month follow-up, suggesting the patient population may differ from the present study in their neural response to tACS. Further investigation of the long-term effect of different multiple-session, multiple-day stimulation protocols at the individual level may help improve target engagement.

On a much shorter time scale, 10 Hz-tACS shifted the peak frequency of whole-brain functional activity toward 10 Hz on Day 5 of the treatment week. This observation replicates our finding in a previous clinical trial using 10 Hz-tACS to treat auditory hallucinations in people with schizophrenia^49^. In addition, tACS also reduced the level of left-right frontal connectivity at 10 Hz on Day 5, which may be explained by synchronized intra-hemisphere stimulation disrupting inter-hemisphere synchronization.

The differentiation between alpha power and functional connectivity results suggests that the amplitude of alpha oscillations and the coordination between alpha oscillations across different brain regions reflect different neural dynamic processes. Computational modeling work indicated that the amplitude and frequency of local oscillations strongly depend on the interaction between local excitatory and inhibitory neuronal populations^75–77^, whereas long-range coordination (i.e., functional connectivity) depends on phase alignment between local oscillatory processes following general dynamical systems principles irrespective of the specific mechanisms of local rhythmogenesis^78–81^. In this light, we may consider alpha power as an indicator of local synchronization and functional connectivity as an indicator of long-range network synchronization between local alpha oscillators. Strengthening of local synchronization does not imply, and may even be *antagonistic* to, strengthening of long-range synchronization. It has been suggested that the inhibitory gating function of alpha oscillation decreases functionally connectivity^82^. Moreover, alpha-tACS has been shown to either increase functional connectivity^83–85^ or decrease functional connectivity^86^ in a region-specific manner, suggesting a complex relation between the strengthening of local oscillation and long-range coordination. Multi-region tACS, as used in the present study, can potentially alter both local synchrony via plasticity and long-range frontoparietal network synchrony via inphase application of currents (Fig. 3), leading to a complex interplay between alpha power changes and functional connectivity changes.

Importantly, 10 Hz tACS significantly reduced participants’ general psychopathology and depression in the short time scale, and symptom reduction was significantly correlated with changes in brain functional connectivity from baseline to Day-5 of the treatment week. Although our stimulation pattern (Fig. 3) was designed for the treatment of auditory hallucinations, it overlaps with a typical design for the treatment of major depressive disorder (MDD). For example, 10 Hz repeated TMS at left dlPFC is an FDA-approved treatment for depression^87,88^, and more recently, 10 Hz tACS at bilateral dlPFC was also shown to improve symptoms in people with MDD^46^. In the present study, the increase of peak frequency of global functional connectivity was significantly correlated with the reduction of both general psychopathology and depression in particular. In our previous clinical trial^49,51^, 10 Hz tACS also induced an increase in peak frequency of global functional connectivity in participants, where we did not observe a concurrent decrease in general psychopathology. The lack of symptom reduction in the previous study was likely due to a floor effect, i.e., baseline general psychopathology was lower (23.63 ± 3.34) in the previous study^51^ when compared to the present study (36.5 ± 11.5). In addition to the peak frequency change, we also observed a reduction in left-right frontal functional connectivity, which was significantly correlated with improvement of general psychopathology. Elevated frontal functional connectivity has been observed in people with MDD^89^, which correlated with symptom severity^48^ and dysphoria^90^. The stimulation protocol of the present study was designed to increase functional connectivity between dlPFC and TPJ within the left hemisphere. Increasing intra-hemisphere connectivity may be an efficient way to disrupt hyperconnectivity between frontal regions across hemispheres. Thus, the present stimulation protocol may serve as a good alternative to bifrontal tACS protocol used in previous studies for the treatment of MDD^46,48^.

We found no significant evidence that 10 Hz-tACS at dlPFC and TPJ is an effective treatment for auditory hallucinations in schizophrenia. This observation is consistent with our previous clinical trial^51^, where we did not observe a significant decrease in AHRS in the tACS group compared to the sham group. Altered alpha oscillations have been observed in people with schizophrenia in terms of lower frequency^33^, reduced power^17,28,34,35^, shifted topography^39^, and decreased functional connectivity^29^. The lack of clinical efficacy of the present protocol in treating auditory hallucinations may have several explanations. First, schizophrenia is a complex disorder, where alpha oscillations are involved in a wide range of symptoms that are non-specific to auditory hallucinations. Our observation of a reduction in general psychopathology supports this idea. Second, our spatial targeting may not be sufficiently specific. Cortical activation during auditory hallucination has greater individual variability than subcortical activation^11^. Individualized spatial targeting may yield a better result. Finally, alpha enhancement alone may not be sufficient to reduce auditory hallucinations, as dysregulation of high-frequency oscillations (e.g., gamma)^7,8^ has also been implicated in schizophrenia. Overall, our findings indicate that alpha oscillations do not represent an effective target for treatment-resistant auditory hallucinations, but rather, a more suitable target for other symptom clusters in people with psychosis.

There are several limitations of this work. First, the cross-over design of the study may not be ideal in light of the long-time scale effect of 10-Hz tACS on alpha oscillations. When designing the study, we did not anticipate that post-stimulation alpha may increase gradually over months, which extended beyond the re-randomization period. Thus, the treatment phase and the maintenance phase of the study were not independent. Second, maximal electric field strength was more central than the electrode location at dlPFC and TPJ. Our results show that alpha power increase was correlated with electric field strength, which is maximal in between, rather than at, dlPFC and TPJ. Future design may optimize electrode placement based on the location of peak field strength. Another limitation is that the resting EEG was always recorded before, rather than after, each stimulation session, including on Day 5 of the treatment week. As a consequence, the changes in EEG from Day 1 to Day 5 reflects the effect of only 4 days of stimulation, which may have led to an underestimation of the effect of the full five-day treatment. The decision to record EEG before tACS/sham was primarily based on the concern that resting EEG right after the Day 5 stimulation session may mainly reflect the acute effect of a single session tACS rather than a complete five-day treatment. Additional consideration includes the effect of prolonged tACS on EEG electrode impedance, which may lead to artifactual changes in EEG in the tACS group. The present procedure reduces such confounding factors in interpreting the EEG findings at the expense of underestimating treatment effects. Moreover, this pilot study has a small sample size. Further validation of the present findings in larger sample will be necessary.

To conclude, 10-Hz tACS significantly alter alpha oscillations in patients with schizophrenia with treatment-resistant auditory hallucinations. tACS treatment did not significantly improve auditory hallucinations but decreased general psychopathology and depression. Symptom improvement was significantly correlated with changes in alpha-band functional connectivity. Our findings suggest that alpha-tACS may represent an effective treatment for symptoms that are non-specific to auditory hallucinations. Further, the study provided initial evidence that alpha-tACS may serve as a transdiagnostic treatment for depression.

## Supplementary information

Supplementary Table S1
