# Supplementary material for: Alpha transcranial alternating current stimulation reduces depressive symptoms in people with schizophrenia and auditory hallucinations: a double-blind, randomized pilot clinical trial
Source: Schizophrenia (Heidelb). 2022 Dec 24;8(1):114. doi: 10.1038/s41537-022-00321-0 (PMC9789318; doi:10.1038/s41537-022-00321-0)
Supplement: Supplementary file 1 — Supplementary Table S1 [file 41537_2022_321_MOESM1_ESM.docx]

|  | **Kolmogorov-Smirnov Test** | **Shapiro-Wilk Test** | **Levene’s Test** |
| --- | --- | --- | --- |
| **AHRS** | D = 0.20  p = 6.23 × 10^-5^ * | W = 0.97  p = 0.0070 * | F_3, 121_ = 2.11  p = 0.102 |
| **HPSVQ** | D = 0.22  p = 1.05 × 10^-5^ * | W = 0.99  p = 0.94 | F_3, 121_ = 8.74  p = 2.72 × 10^-5^ * |
| **PANSS** |  |  |  |
| - total | D = 0.27  p = 3.49 × 10^-5^ * | W = 0.94  p = 0.0017 * | F_3, 71_ = 2.82  p=0.045 * |
| - Positive symptoms | D = 0.10172  p = 0.39 | W = 0.9784  p = 0.23 | F_3, 71_ = 1.46  p = 0.23 |
| - Negative symptoms | D = 0.15  p = 0.066 | W = 0.98  p = 0.49 | F_3, 71_ = 0.51  P = 0.68 |
| - General psychopathology | D = 0.17  p = 0.018 * | W = 0.95  p = 0.0060 * | F_3, 71_ = 4.49  p = 0.0061 * |
| - Hallucinations | D = 0.32  p = 3.79 × 10^-7^ * | W = 0.97  p = 0.063 | F_3, 71_ = 0.22  p = 0.88 |

**Table S1. Diagnostic tests of residual normality and homoscedasticity**. Diagnostic statistics are shown for the residuals of the linear mixed effect model for each symptom measure (see F-test results in Table 4 of the main text). * p < 0.05.
